# Structural Insights of *Shigella* Translocator IpaB and Its Chaperone IpgC in Solution

**DOI:** 10.3389/fcimb.2021.673122

**Published:** 2021-04-29

**Authors:** Mariana L. Ferrari, Spyridoula N. Charova, Philippe J. Sansonetti, Efstratios Mylonas, Anastasia D. Gazi

**Affiliations:** ^1^ Unité de Pathogénie Microbienne Moléculaire, Institut Pasteur, Paris, France; ^2^ INSERM U1202, Paris, France; ^3^ Institute of Molecular Biology and Biotechnology, Foundation for Research and Technology – Hellas (IMBB-FORTH), Heraklion, Crete, Greece; ^4^ Collège de France, Paris, France; ^5^ UtechS Ultrastructural Bio-Imaging (UBI), Institut Pasteur, Paris, France

**Keywords:** type III secretion (T3S), type III translocator, small angle x-ray scattering, IpgC chaperone, IpaB translocator, Shigella flexneri

## Abstract

Bacterial Type III Secretion Systems (T3SSs) are specialized multicomponent nanomachines that mediate the transport of proteins either to extracellular locations or deliver Type III Secretion effectors directly into eukaryotic host cell cytoplasm. *Shigella*, the causing agent of bacillary dysentery or shigellosis, bears a set of T3SS proteins termed translocators that form a pore in the host cell membrane. IpaB, the major translocator of the system, is a key factor in promoting *Shigella* pathogenicity. Prior to secretion, IpaB is maintained inside the bacterial cytoplasm in a secretion competent folding state thanks to its cognate chaperone IpgC. IpgC couples T3SS activation to transcription of effector genes through its binding to MxiE, probably after the delivery of IpaB to the secretion export gate. Small Angle X-ray Scattering experiments and modeling reveal that IpgC is found in different oligomeric states in solution, as it forms a stable heterodimer with full-length IpaB in contrast to an aggregation-prone homodimer in the absence of the translocator. These results support a stoichiometry of interaction 1:1 in the IpgC/IpaB complex and the multi-functional nature of IpgC under different T3SS states.

## Introduction

Gram-negative bacteria have evolved a specialized secretion mechanism that allows the communication with higher organisms, resulting either in pathogenesis or symbiosis (Coombes, 2009; Tampakaki et al., 2010; Puhar and Sansonetti, 2014). These multi-component nanomachines, called Type III Secretion System (T3SS), are integrated into the two bacterial membranes and serve as main conductor channels for substrate selection and secretion (Portaliou et al., 2016). T3SSs are encoded by genes tightly packed in the bacterial chromosome that are usually located inside pathogenicity islands (PAIs), or in virulence plasmids as in the case of *Shigella* (Bajunaid et al., 2020). They have evolved from the bacterial flagellum and later diversified into 7 to 8 host-cell adapted systems (Abby and Rocha, 2012; Gazi et al., 2012). *Shigella*, the causing agent of shigellosis, a life-threatening form of bacillary dysentery, uses a T3SS to promote its own uptake by human intestinal epithelial cells and then move inside the eukaryotic cytoplasm. After that, the bacteria multiply and spread into neighboring cells (Bajunaid et al., 2020).

The T3SS pathway allows *Shigella* to not only secrete proteins to the extracellular milieu but also to directly translocate them into the host cell cytoplasm. This is achieved through the formation of a pore in the eukaryotic cell membrane and the subsequent docking of the T3S-apparatus (T3SA) on it. The translocation pore in *Shigella* is formed by two proteins: Invasion plasmid antigens B and C (IpaB and IpaC) (Blocker et al., 1999; Matteï et al., 2011), which are also T3S substrates themselves. A tightly- controlled multi-step folding pathway is followed, comprising i) translation of the nascent polypeptides, ii) maintenance in the bacterial cytoplasm in a secretion competent state, iii) delivery to the secretion apparatus, iv) traversing through the narrow T3S needle channel, v) maintenance on the distant end of the machinery (tip of the needle), vi) insertion and polymerization into the host cell membrane and rearrangement of the translocation pore to efficiently dock the T3S needle (Olive et al., 2007; Stensrud et al., 2008; Cheung et al., 2015; Barta et al., 2018; Russo et al., 2019).

IpaB is further characterized as one of the main players in promoting *Shigella* pathogenicity: it is central for host cell invasion through secretion regulation and host cell sensoring, phagosome escaping and macrophage cell death induction (Picking and Picking, 2016). For its multifunctional properties, many studies have focused on this protein as a potential key ingredient of a future vaccination mix against shigellosis (Chen et al., 2015; Heine et al., 2015; Turbyfill et al., 2018).

IpgC, a small (15 kDa) hydrophilic protein, was identified as the IpaB and IpaC cognate chaperone by interacting independently with both of them (Ménard et al., 1994; Page et al., 1999). IpgC, IpaB and IpaC are all produced from the same operon by adjacent genes following this exact order. The operon continues to the production of IpaD, the protein building the pentameric tip of the T3S needle (Epler et al., 2012) and ends up with the production of IpaA, the T3SS effector that interacts with host’s vinculin and modulates the entry of *Shigella* into epithelial cells (Tran Van Nhieu et al., 1997). Therefore, synchronized production of these proteins is a vital strategic step. IpgC is required both to block the premature association of IpaB to IpaC and to maintain the translocators in a secretion competent state (Ménard et al., 1994). IpaB and IpaC are aggregated and degraded in the cytoplasm of a non-polar *ΔipgC Shigella* mutant strain, indicating a stabilizing function of IpgC on the translocators (Ménard et al., 1994; Page et al., 1999). It has also been shown that IpaB oligomers disrupt liposomes *in vitro* (Senerovic et al., 2012; Dickenson et al., 2013), suggesting an additional function of IpgC in preventing a premature association of IpaB to the bacterial inner membrane. Their secretion, following detection of host cell proximity, leads to the release of IpgC in the bacterial cytoplasm (Mavris et al., 2002). In parallel, secretion of OspD1, that sequesters MxiE in the T3SS inactive state, results in higher MxiE levels in the bacterial cytoplasm. MxiE, an AraC-like transcription activator, associates then to IpgC and induces the expression of the second wave of T3SS effectors (Mavris et al., 2002; Pilonieta and Munson, 2008).

**Figure 1:**
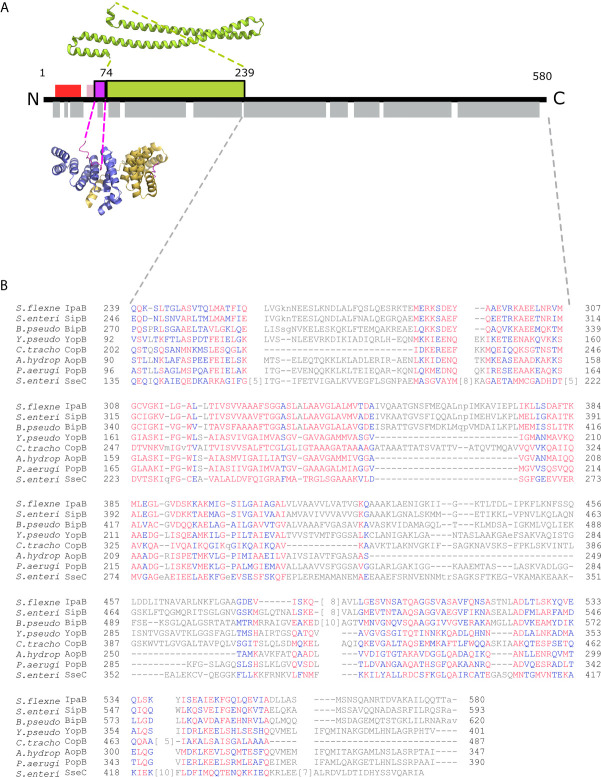
IpgC/IpaB available structural information. **(A)** A schematic representation of the IpaB full-length protein and the available structural information. The long coiled-coil domain of IpaB (green rectangle, residues 74 - 239) is the only known domain in atomic resolution along with the Chaperone Binding Domain (CBD, magenta rectangle, residues 60-72). The CBD domain was found in extended conformation interacting with the major groove of one IpgC molecule (cartoon representation, blue monomer). See text for details. Other segments of IpaB were proposed as being implicated in the interaction with IpgC: a second CBD (red rectangle) at the N-terminal of IpaB; and an extension of the first CBD (pink rectangle). Grey rectangles on bottom of IpaB sequence represent predicted α-helices. **(B)** Constraint-based Multiple Alignment, COBALT (Papadopoulos and Agarwala, 2007). Part of the alignment, focusing on the C-terminal part of major translocators from T3S systems of different origin, is presented. Multiple sequence alignment columns with no gaps are colored. Higher conserved columns in red, less conserved ones in blue and non-conserved in grey. Accession numbers for the protein sequences used are provided in *Materials and Methods* section *Modeling of the IpgC/IpaB Complex*.

Atomic resolution information on IpaB is limited to a soluble coiled-coil domain (Barta et al., 2012; Barta et al., 2018) located after the 13-residues long Chaperone Binding Domain (CBD) (**Figure 1A**), that has also been co-crystallized bound to the IpgC chaperone (Lunelli et al., 2009). IpaB full-length was found to be unstable in the absence of IpgC, while its heterologous overproduction in *Escherichia coli* was only possible in the presence of the chaperone (Ménard et al., 1994; Page et al., 1999; Lokareddy et al., 2010).

The IpgC chaperone was found to be homodimeric, although two different dimerization modes have been observed in two different crystal forms, one being asymmetric (Lunelli et al., 2009; Barta et al., 2010). This observed dimerization led to the assumption that IpgC binds IpaB also as a dimer in a 2:1 stoichiometry (Lokareddy et al., 2010). However, data supporting the 1:1 stoichiometry for the IpgC/IpaB complex have also been reported (Birket et al., 2007; Adam et al., 2012). In the absence of a high-resolution atomic structure of the full-length protein IpaB and its complex with IpgC, their association still remains elusive.

In this study we employed biochemical and biophysical methods to gain structural information on the solution structure of IpgC and its association to IpaB. Small Angle X-ray Scattering (SAXS) is a low-resolution method that provides robust insights on the polymerization of particles under various concentrations in solution, information that is usually omitted or masked by the tight particle packing in the crystal lattice. Our results validate the dimeric form of IpgC when isolated in solution as well as present the first low-resolution model of the full-length IpgC/IpaB complex that reflects its native organization when inside the bacterial cytoplasm.

## Materials and Methods

### Preparation of Protein Samples

Purified IpaB and IpgC proteins from *Shigella flexneri* 5a (M90T) were obtained as previously described (Senerovic et al., 2012). Briefly, *ipaB* and *ipgC* were cloned in the expression vectors pET21a(+) and pET28a(+) respectively, (plasmids pMK101 and pMK001, respectively, in (Lokareddy et al., 2010)), and co-expressed in *E. coli* BL21(DE3) cells. The N-terminal His-tagged IpgC and its complex with IpaB were purified first with a HisTrap HP column (GE Healthcare) using standard procedures followed by size exclusion chromatography on a High Load 16/60 Superdex 200 prep grade gel filtration column (GE Healthcare) in 20 mM HEPES (pH 7.4), 100 mM NaCl. The gel filtration column was calibrated in three different runs using protein standards (GE Healthcare-Life Sciences gel filtration calibration LMW and HMW kits) in the same buffer used for IpgC and IpaB (20 mM HEPES pH 7.4, 100 mM NaCl buffer). Hydrodynamic radius (R_h_) or apparent Stokes radius (R_s_) and apparent Molecular Weight (MW) of IpgC/IpaB were calculated based on the known standard as described in (La Verde et al., 2017). Briefly, Blue Dextran (~2000 kDa) was loaded in a separate run, while protein standards were mixed in two different combinations, in order to avoid peak overlaps. Ferritin (MW = 440kDa; R_s_ = 61Å), Conalbumin (MW = 75kDa) and Carbonic anhydrase (MW = 29kDa; R_s_ = 23,5Å) were mixed in recommended concentrations and loaded to the gel filtration column for the first run while, Aprotinin (MW = 6,5 kDa; R_s_ = 13,5Å), Ribonuclease A (MW= 13,7kDa; R_s_ = 19,4Å), Ovalbumin (MW = 44 kDa; R_s_ = 33,5Å) and Aldolase (MW = 158 kDa; R_s_ = 48,1Å) were mixed together for the second run. Molecular Weight (MW) and Stokes radii (R_s_) were provided by the GE Healthcare technical data sheet at that time. The values of R_s_ have been reported for a large number of proteins (Uversky, 1993; La Verde et al., 2017).

### SAXS Data Collection and Processing

Two different data sets were collected at 10 °C for various IpgC/IpaB concentrations in 20 mM HEPES (pH 7.4), 100 mM NaCl, ranging from 0.58 to 5.48 mg/ml at the BM29 BioSAXS beamline of the ESRF synchrotron (Pernot et al., 2010; Pernot et al., 2013). Five data sets were collected for IpgC corresponding to three different sample preparations (concentrations ranging from 0.2 to 7.8 mg/ml). Samples were either kept on ice or were flash frozen and kept in liquid nitrogen until their arrival to the experimental site. Samples were then thawed on ice, centrifuged and the absorption at 280nm of the supernatant was monitored with Nanodrop. Dilutions were prepared with the same buffer solution and samples were loaded using the beamline’s automated system to a 1.8 mm diameter quartz capillary with a few tens of micron wall thickness using continuous flow during data collection. The SAXS data were recorded using a Pilatus 1 M detector at a sample to detector distance of 2.84 m, covering the range of momentum transfer 0.003<q<0.45 Å^−1^ (q= 4πsin(θ)/λ where 2θ is the scattering angle and λ = 0.9199 Å is the X-ray wavelength). To evaluate radiation damage, ten successive 10 sec exposures of each sample were recorded. Data collection, processing and analysis were performed in an automated manner using the dedicated beamline software BsxCuBE. The PRIMUS software (Konarev et al., 2003) was used for evaluation of the radiation damage and sample monodispersity, buffer subtraction, extrapolation to zero concentration, data scaling and merging. Estimation of the radius of gyration (R_g_) and of the forward scattering intensity I(0) (proportional to the number of electrons in the particle) was also performed with PRIMUS using the Guinier approximation (Guinier, 1939; Guinier and Fournet, 1955). Molecular weights (MW) were estimated from the I(0) by comparing with a 5 mg/ml BSA solution. The indirect Fourier transform package GNOM (Svergun, 1993) was used for the evaluation of the maximum particle diameter (D_max_) and the calculation of the pair distribution [P(r)] function as well as the R_g_. The integrity of the stock samples was verified after the experiment with SDS-PAGE (**Supplementary Figure 1**).

### Modeling of the IpgC Dimer

The scattering patterns of the high-resolution dimeric models of IpgC (PDB IDs: 3GYZ, 3KS2) (Lunelli et al., 2009; Barta et al., 2010) were computed and compared to the experimental data using the program CRYSOL (Svergun et al., 1995). Because a significant portion of the protein is not present in the crystal structures, we used the program CORAL of the ATSAS package (ATSAS, RRID : SCR_015648) (Petoukhov et al., 2012) to account for the electron density of the missing residues by adding dummy residues.

### Modeling of the IpgC/IpaB Complex

All handling of PDB files was done using UCSF Chimera (UCSF Chimera, RRID : SCR_004097) (Pettersen et al., 2004). The known crystal structure of the IpaB coiled coil domain, covering the amino acid residues 74 to 239, was retrieved from the Protein Data Bank (PDB ID: 5WKQ). The Chaperon Binding Domain (CBD) domain (residues 60 – 72) is located directly upstream of the coiled coil domain in IpaB with only one intervening residue. The CBD and the coordinates of the IpgC chaperone bound to it were extracted from the PDB entry 3GZ1. The amino acid sequence of IpaB was introduced as a query in the fold recognition server PHYRE (Phyre, RRID : SCR_010270) (Kelley et al., 2015). A total of 162 residues out of the 357 used were modeled with a 99.7% confidence based on the *Aeromonas hydrophila* AcrH/AopB chaperone - translocator complex structure (PDB ID: 3WXX) (Nguyen et al., 2015). Similar results were obtained with the HHpred prediction server (Bioinformatics Toolkit, RRID : SCR_010277) (Zimmermann et al., 2018) (**Supplementary Figure 2**). Constraint-based Multiple Alignments (COBALT) (Papadopoulos and Agarwala, 2007) are also indicative of the similarity of IpaB to AopB and other translocators (**Figure 1B**). Gap penalties used for COBALT were -11 and -1 (opening & extension), end-gap penalties -5 and -1 (opening & extension). RPS BLAST, ‘Find conserved columns and recompute’ and ‘Use of query clusters’ options were on. Protein BLAST threshold on 0.003. 4-mer based sequence similarity. Maximum allowed distance between distances in a cluster at 0.8. Regular alphabet was used. Accession numbers for sequences analyzed in **Figure 1B** are: ADA76866.1 IpaB (plasmid) [*Shigella flexneri* 2002017], AAA75169.1 SipB [*Salmonella enterica* subsp. enterica serovar Typhimurium str. SL1344], ABO28796.1 BipB [B*urkholderia pseudomallei* Pasteur 52237], AAA72321.1 yopB [*Yersinia pseudotuberculosis*], CCP29276.1 putative type III secretion system membrane protein [*Chlamydia trachomatis* IU888], AAR26341.1 AopB [*Aeromonas hydrophila*], AAG05097.1 translocator protein PopB [*Pseudomonas aeruginosa* PAO1], CAA12187.1 SseC [*Salmonella enterica* subsp. enterica serovar Typhimurium]. No high-resolution structures displayed significant homology with the extreme C-terminal part of IpaB. The AcrH was also used as a template to position IpgC relative to IpaB and model the IpgC N-terminal helix. The relative positioning of the atomic models and the remaining residues (~25% of the total His-tagged IpgC and IpaB) were modeled with CORAL (Petoukhov et al., 2012) and EOM (Bernadó et al., 2007; Tria et al., 2015) based on the SAXS data. All SAXS data and models presented in the manuscript are accessible from SASBDB (Kikhney et al., 2020).

## Results

### Basic Hydrodynamic Observations From Gel Filtration

**Figure 2:**
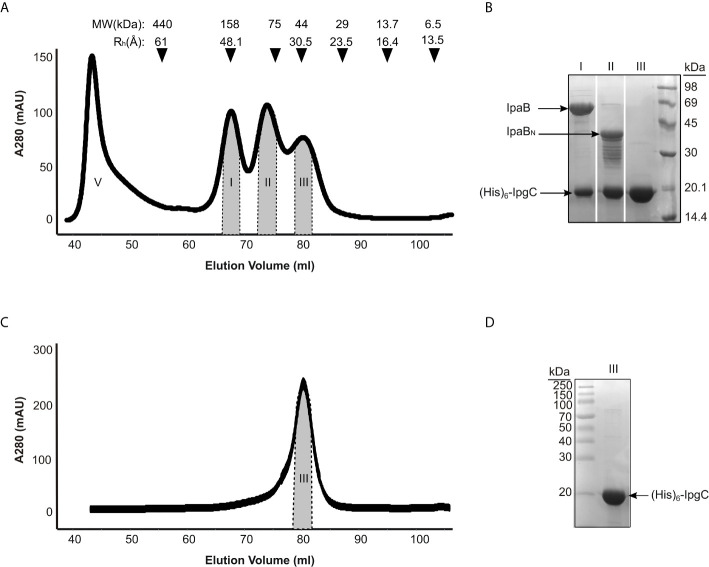
IpgC/IpaB available structural information and hydrodynamic parameters. **(A)** Chromatograph of Size Exclusion analysis on the elution fractions of the IpgC/IpaB complex collected following the metal affinity purification step. Four peaks are observed; V: Void Volume; I: The IpgC/IpaB peak as judged by the SDS-PAGE analysis in **(B)**; II: A proteolytic form of IpaB in complex with IpgC; and III: IpgC alone. On top of the graph the apparent Molecular Weights (MW) and the Hydrodynamic Radii (R_h_) of the molecular markers used to calibrate the size exclusion chromatography column are shown. **(B)** SDS-PAGE analysis of the various peaks in A after their collection and concentration. **(C)** Chromatograph of Size Exclusion analysis on the elution fractions of the IpgC collected following the metal affinity purification step. **(D)** SDS-PAGE analysis of the IpgC peak in **(C)**.

Three main peaks were typically detected in size exclusion chromatography of co-expressed IpgC/IpaB after the metal affinity chromatography step (**Figure 2A**). Analysis by SDS-PAGE (**Figure 2B**) showed that only the first one contains the full length IpaB along with IpgC (**Figures 2A, B**, peak I). This peak represents a particle with a ~48 Å hydrodynamic radius according to known molecular markers. The apparent molecular weight of the particle (in case of a spherical particle) was calculated at ~158 kDa consistent with previous findings (Birket et al., 2007). The second peak contains IpgC and a 40 kDa band. Peptides isolated from this band were identified by mass spectrometry to belong mostly to the N-terminal part of IpaB (**Supplementary Table 1**). Some peptides of the C-terminal part were also identified but only in trace amounts, probably as a result of contamination with full-length IpaB. Because of the polydispersity of this peak (**Figures 2A, B**, peak II), it was not further considered for analysis. The third peak elutes at an apparent MW of 42 kDa and corresponds to the His-tagged IpgC dimer (**Figures 2A, C**, peak III).

### Basic Observations From SAXS Data

**Figure 3:**
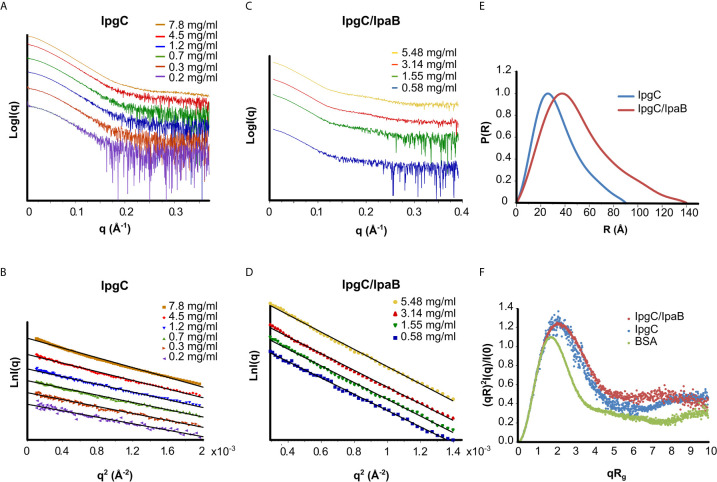
SAXS analysis of the IpgC **(A, B, E, F)** and IpgC/IpaB complex **(C–F)**. **(A, C)** SAXS Intensity profiles (in logarithmic scale) for six concentrations of IpgC **(A)** and four concentrations of the IpgC/IpaB complex **(C)**. **(B, D)** Guinier plots linearity indicates monodispersity for the IpgC/IpaB complex **(D)** and aggregation for the higher concentrations of IpgC **(B)**. **(E)** Normalized pair distance distribution functions P(r) for IpgC and IpgC/IpaB. **(F)** Normalized Kratky plots of IpgC and the IpgC/IpaB complex (a globular, well folder protein, BSA, is also shown for comparison).

The IpgC particle (peak III in **Figures 2A–D**) (R_g_ = 27 ± 1 Å, D_max_ = 90 Å) exhibited significant but reversible aggregation at higher concentrations (**Figures 3A, B** **)**. At the lowest concentrations, the MW of IpgC was estimated from the Guinier approximation at ~37kDa, consistent with a dimer (calculated MW=39kDa), indicating that this is the smallest unit in which free IpgC is found in solution. The Dimensionless Kratky plot (Durand et al., 2010) (**Figure 3F**) shows a prominent and well-defined peak albeit wider than that of a globular protein, such as BSA, hinting to higher anisometry and flexibility.

Only weak concentration effects were observed for the IpgC/IpaB complex (peak I in **Figure 2A**), as evidenced by the linearity of the Guinier regions and the stability of the R_g_ across different concentrations (**Figures 3C, D**), indicating both absence of aggregation and stability of the complex. The radius of gyration of the particle was estimated at 39 ± 1 Å, with a maximum diameter of 140 Å, derived, respectively by the Guinier approximation and the pair distribution function, P(r) (**Figures 3D, E** **)**, which also suggests a moderately elongated shape in solution. Very similar to IpgC, the dimensionless Kratky plot (**Figure 3F**) shows a prominent but slightly wide peak, indicating that the protein complex has a well-defined shape, but it is elongated and exhibits some flexibility. The absence of concentration effects suggests that the IpgC/IpaB complex is stable, monodisperse and exists as one species with specific stoichiometry in solution, because if different types of complexes coexisted, one would expect a concentration-dependent behavior. Nevertheless, the estimation of the MW of the complex from the Guinier approximation presented a challenge. IpaB, which is more than three times larger than IpgC, contains only one tryptophan and a few tyrosines, reducing the reliability of concentration measurements by absorption at 280nm (to which IpgC also contributes). Intriguingly, the ratio of the molecular weights for 1:1 (82kDa) and 2:1 (101kDa) stoichiometries of the IpgC/IpaB complex to their respective extinction coefficients $\left(A_{280nm}^{1mg/ml}\right)$ is almost equal, in turn producing estimated MWs from the Guinier approximation equally close to their respective expected MW values. In fact, the MW determination by use of a static light scattering detector coupled with a UV detector that led Lokareddy et al. (2010) to propose a 2:1 stoichiometry could very well have been a 1:1 complex, equally compatible with their data. For this purpose, the Bayesian inference approach (Hajizadeh et al., 2018) of PRIMUS was employed, giving a MW of 83kDa. Previous observations (Birket et al., 2007; Adam et al., 2012) as well as structural information from homologous proteins (Nguyen et al., 2015) suggest that the IpgC/IpaB complex is in a 1:1 stoichiometry.

### The Dimeric Form of IpgC Is Predominantly Symmetric in Solution

**Figure 4:**
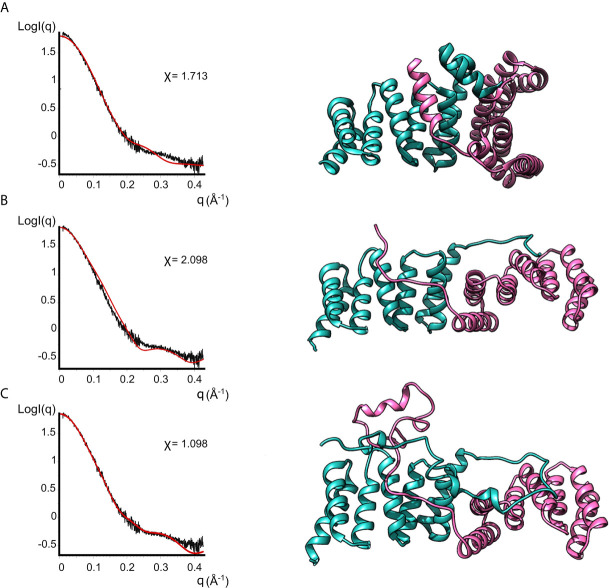
Models of IpgC dimers (right panels) and the corresponding fits to solution SAXS data (left panels). Model **(A)** is the asymmetric IpgC dimer (PDB ID: 3GZ1). Model **(B)** is the symmetric IpgC dimer (PDB ID: 3KS2). Model **(C)** is the symmetric IpgC dimer where the residues not present in the crystal structure were modeled to be compatible with the SAXS pattern.

None of the crystal structures available in the PDB are in good agreement with the experimental data, as evidenced by the bad fits of the calculated scattering patterns to the experimental SAXS data (**Figure 4** and **Supplementary Table 2**). This can be attributed to the fact that the crystal structures are missing residues compared to the full-length protein plus the His-Tag present in our IpgC construct. For such a small protein, these residues constitute a significant portion of the total mass of the protein (~20%) and the contribution of their electron density to the scattering pattern cannot be ignored.

To better fit the experimental SAXS data, CORAL was used to take into account the contribution of the tag (**Figure 4**). The crystallographic dimers were treated as rigid bodies and the missing residues were added to account for the extra electron density. Only models based on the symmetric dimer were in good agreement with the experimental data (**Figure 4C**, χ=1.098). Even without adding the missing residues, the theoretical scattering pattern of the symmetric model (Barta et al., 2010) follows the general trend of the experimental data while the asymmetric models (Lunelli et al., 2009) fail to reproduce features of the scattering pattern, more specifically the peak at q≈0.3Å^-1^. This indicates that in solution the protein is predominantly found in a symmetric dimeric arrangement at low concentrations. The higher order oligomers likely also involve interactions similar to those found in the asymmetric crystallographic dimer, since the interaction interface is different between the symmetric and the asymmetric dimer.

### Structure of the 1:1 IpgC/IpaB Complex in Solution

**Figure 5:**
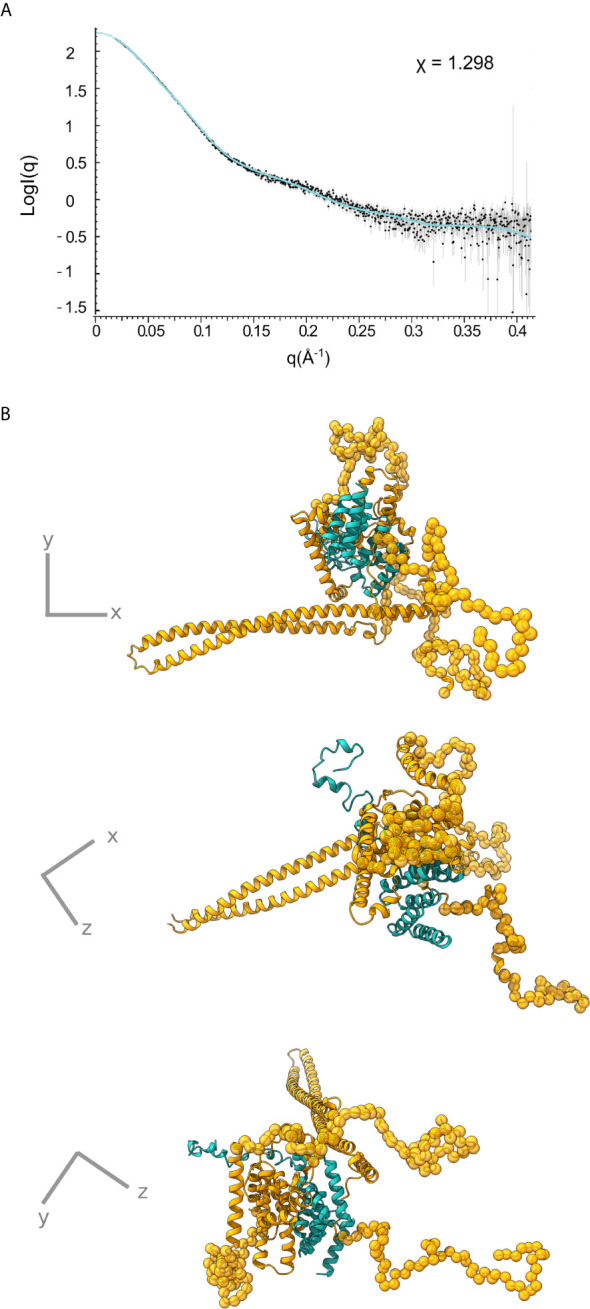
IpgC/IpaB SAXS model. **(A)** Fit of the theoretical SAXS pattern calculated from the model in **(B)** to the experimental SAXS data. **(B)** Three perpendicular representations of the IpgC/IpaB SAXS model. IpaB is depicted in yellow-gold and IpgC in turquoise. Residues where high resolution information is available (crystal structure or homology model) are shown in cartoon representation while spheres represent dummy residues.

Only the N-terminal long coiled coil domain and the CBD peptide of IpaB have been structurally resolved in high resolution. Additionally, the middle part of the IpaB sequence exhibits homology (**Figure 1B**) with a crystallographically determined region of translocator AopB from *Aeromonas hydrophila* (Nguyen et al., 2015). Conveniently, AopB was solved complexed with its chaperone AcrH, which shows a structure extremely similar to IpgC. The AopB/AcrH heterodimer shows extensive interaction interfaces between the two proteins, not limited to the CBD peptide. Moreover, the tightness of the complexation precludes the possibility of chaperone dimerization (either symmetric or asymmetric) because the translocator occupies the interfaces implicated in chaperone dimerization (Nguyen et al., 2015). The SAXS analysis also shows that the interaction is very stable, indicating that the interaction of IpaB with IpgC disrupts the dimerization/oligomerization of IpgC. Thus, a homology model of the central domain of IpaB based on AopB and the IpgC structure docked on IpaB (including CBD) was produced. This model together with the coiled coil N-terminal IpaB domain and a predicted C-terminal α-helix, were used as rigid bodies free to move relative to each other, while missing residues of both IpaB and IpgC were modeled in a manner similar to the IpgC dimer model (**Supplementary Table 2**). A representative model and the corresponding fit of the model to the experimental SAXS pattern (χ=1.298) are shown in **Figure 5** (see also **Supplementary Video 1**). The complex consists of a central “blob” where the bulk of the protein mass resides, including the IpgC/IpaB interaction, from which the long coiled-coil N-terminal domain of IpaB protrudes, producing a “pear-shaped” overall structure. The elongated nature of the structure likely explains the physical properties of the complex, i.e. the earlier than expected elution from a Size Exclusion Chromatography column.

## Discussion

The IpgC/IpaB complex was found to be highly stable in solution (Birket et al., 2007). This strong interaction of the IpgC chaperone to IpaB possibly prevents the interaction of IpaB to the second T3S translocator protein already present in the bacterial cytoplasm, IpaC, and maintains IpaB in a secretion competent folding state (Ménard et al., 1994; Page et al., 1999), yet a function in preventing IpaB to prematurely bind the bacterial membrane cannot be excluded (Dickenson et al., 2013). However, the molecular details of the IpgC/IpaB interaction still remain elusive.

In previous studies, IpgC was found to be dimeric in crystals (Lunelli et al., 2009; Barta et al., 2010). Our Size Exclusion Chromatography results and the SAXS analysis also support this view: IpgC was found to be dimeric with the two monomers arranged probably in a symmetric way. Regarding IpaB, only 30% of its sequence corresponds to known atomic coordinates, namely the long coiled-coil domain present in the N-terminal of IpaB (Barta et al., 2012; Barta et al., 2018) and a small peptide sequence, the Chaperone Binding Domain (CBD), located just upstream of the coiled coil domain that binds to the major groove of the IpgC chaperone (Lunelli et al., 2009). Like IpgC, the IpaB coiled-coil domain was also found to be dimeric in crystals and in solution (Adam et al., 2012). In Size Exclusion Chromatography, the IpgC/IpaB complex migrates with an apparent molecular weight of 158 kDa (hydrodynamic radius, R_h_, of 48.1 Å) that could imply a 2:2 or 2:1 interaction (163 kDa and 101 kDa respectively). However, previous reports suggested an equimolar ratio interaction based on isothermal titration calorimetry, fluorescence polarization and cross-linking studies (Birket et al., 2007; Adam et al., 2012). Sedimentation velocity analysis indicated a molecular weight of around 80 kDa for the IpgC/IpaB complex, with the friction coefficient f/f_0_ of around 1.7 (Birket et al., 2007). This, together with our SAXS data, indicates the highly elongated nature of the IpgC/IpaB complex that explains the Size Exclusion Chromatography results. Considering the previous findings, the stoichiometry for the IpgC/IpaB complex most compatible with our results and the available bibliographic data is 1:1. This is also supported by the structural homology of IpgC/IpaB to the *A. aerophila* AcrH/AopB structure, where the interaction between the chaperone and the translocator is 1:1 (Nguyen et al., 2015).

IpgC is a tetratricopeptide repeat (TPR) protein. TPRs comprise 34-residue motifs that assemble into a helix-turn-helix fold and are usually found in tandem repeats that adopt an extended, right-handed super-helical fold followed by a C-terminal hydrophilic ‘capping-helix’. These domains are usually implicated in interactions with other α-helical domains or with themselves, already known to form asymmetric oligomers (Krachler et al., 2010). Our SAXS experiments are indicative of the reversible self-association nature of IpgC in solution, a typical behavior of TPR domains. In contrast, the IpgC/IpaB complex is a stable, monodisperse complex in solution.

Our SAXS data suggest the presence of a compact, yet elongated IpgC/IpaB particle in solution with the N-terminal coiled-coil domain of IpaB contributing to its longest dimension, extending from the main particle mass. IpgC and the C-terminal IpaB domain comprise the main blob implying that there is possibly a larger buried surface of IpgC in the IpgC/IpaB particle as proposed from the homologous AopB domain. This can also further explain why, in studies where a large portion of the IpaB sequence is deleted, IpgC was found as a dimer in solution (Lunelli et al., 2009; Lokareddy et al., 2010), as more of its TPR α-helices are exposed and free to interact. Except for the CBD domain of IpaB, there is an additional region in the N-terminus of the IpaB sequence that appears to contribute additively to chaperone binding (Lokareddy et al., 2010; Adam et al., 2012). This additional binding site may strengthen the interaction of IpaB with IpgC rather than engage a second IpgC molecule.

Lokareddy and co-workers (Lokareddy et al., 2010) suggested a 2:1 association between IpgC and IpaB based on Multi Angle Laser Light Scattering experiments (MW around 100 kDa), although, as explained, their results are also perfectly compatible with a 1:1 association. They also observed dimerization of IpgC in the presence of smaller IpaB fractions. However, based on the AcrH/AopB structure and on the fact that IpaB possesses a domain homologous to the one in AopB that interacts with its TPR chaperone using an extensive interaction surface, it is possible that smaller IpaB fragments are not enough to hinder the IpgC dimerization in solution. Interestingly, the purported AopB-like dimerization interface of IpaB largely coincides with the 313-346 hydrophobic region of IpaB shown to be the most critical region for IpgC dependency (Ménard et al., 1994), while the N-terminal CBD could act as an IpgC anchor as proposed for the AcrH/AopB case (Nguyen et al., 2015).

IpaB is predicted to be a highly α-helical protein (Psi-PRED, **Figure 1A**) and TPR domains are highly α-helical associated domains that readily change their oligomeric state. The structural plasticity of the TPR proteins, the plethora of dimerization interfaces exhibited by IpgC and homologs from other organisms (Büttner et al., 2008; Job et al., 2010) and the tendency to form higher order soluble oligomers (this study), are properties that support these proteins as key interaction partners bearing multiple roles under various T3SS states.

To summarize, IpgC is produced by the same operon as the translocators IpaB and IpaC, ensuring the synchronized translation of these polypeptides in time and space. Taking together the IpaB and IpaC instability in a *Shigella ΔipgC* mutant (Ménard et al., 1994; Page et al., 1999), the insolubility and toxicity of IpaB and the low solubility of IpaC when expressed alone in *E. coli* (Lokareddy et al., 2010), we conclude that IpgC most probably binds IpaB and IpaC quite early after or during their translation in an equimolar ratio. The fact that both T3SS translocator and effector proteins anchor their N-terminal part to cognate chaperones, probably as soon as their N-terminal polypeptides are produced from the ribosomes, may help to maintain them in a secretion-competent folding state while residing inside the bacterial cytoplasm and until their delivery to the T3SS sorting platform for unfolding. IpgC further protects the transmembrane domain of the translocator by forming a stable 1:1 complex, in a similar way to the AopB/AcrH case. The observed homodimerization of IpgC *in vitro*, when isolated in solution, might be related to a different biological function that follows in time, after the secretion of IpaB and IpaC, like the implication of IpgC in MxiE-dependent transcription regulation (Mavris et al., 2002).

## Data Availability Statement

The datasets presented in this study can be found in online repositories. The names of the repository/repositories and accession number(s) can be found below: https://www.sasbdb.org/, SASDKQ9, SASDKR9.

## Author Contributions

MF and AG have contributed to sample preparation. SC and AG have contributed to data collection. EM and AG have contributed to data analysis. PS provided financial support. All authors contributed to the article and approved the submitted version.

## Funding

The project was funded by the European Research Council (ERC) Grants 232798 and 339579 (PS). MF was supported by the French Medical Research Foundation (SPF20121226366). AG was supported by FP7-PEOPLE PIAPP-GA-2008-217768. SC was funded by E.E. & GSRT action “Support of postdoctoral researchers” (LSI-1808). SC and EM were supported by a grant from the Hellenic Foundation for Research and Innovation (HFRI). We acknowledge UTechS UBI support from the French Government (Agence Nationale de la Recherche, ANR): Programme Investissements d’Avenir France BioImaging (FBI, N° ANR-10-INSB-04-01) and the Investissement d’Avenir programme, Laboratoire d’Excellence “Integrative Biology of Emerging Infectious Diseases” (ANR-10-LABEX-62-IBEID).

## Conflict of Interest

The authors declare that the research was conducted in the absence of any commercial or financial relationships that could be construed as a potential conflict of interest.
