# Supplementary material for: Structural Insights of Shigella Translocator IpaB and Its Chaperone IpgC in Solution
Source: Front Cell Infect Microbiol. 2021 Apr 29;11:673122. doi: 10.3389/fcimb.2021.673122 (PMC8117225; doi:10.3389/fcimb.2021.673122)
Supplement: Supplementary file 1 [file DataSheet_1.pdf]

Supplementary Material

Supplementary Figures and Tables

1. Supplementary Figures

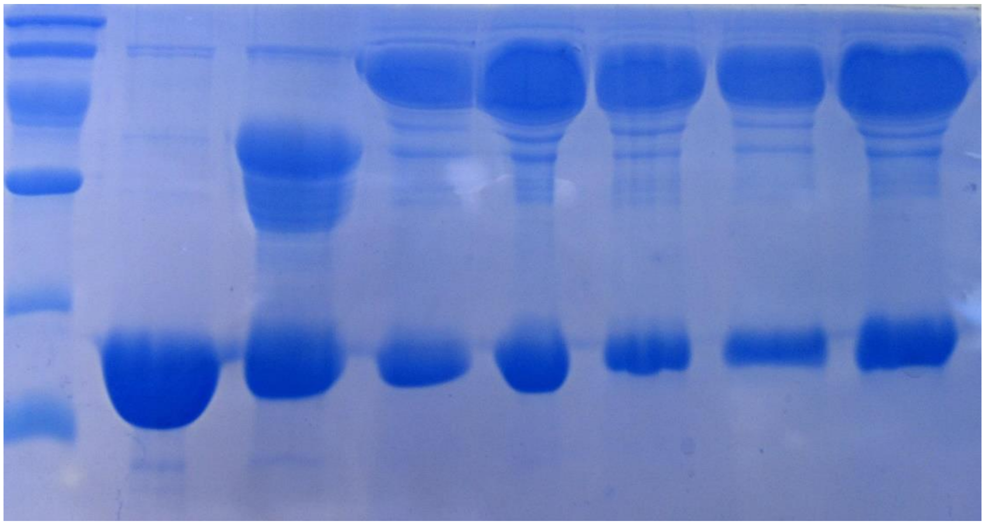

Supplementary Figure 1. SDS-PAGE analysis of the samples used in the SAXS experiments after returning to the home laboratory. From left to right: 1) LMW marker (GE Healthcare): 98 kDa, 69 kDa, 45 kDa, 30 kDa, 20.1 kDa, 14.4 kDa, 2) IpgC, 3) IpgC with IpaB Nter, 4 – 8) IpgC with full length IpaB.

Template alignment | Template 3D Structure | PDBe

3WXX_B AopB; Translocator, Membrane protein-Chaperone complex; HET: MSE; 2.7A {Aeromonas hydrophila}

Probability: 99.51%, E-value: 3e-12, Score: 127.37, Aligned cols: 154, Identities: 19%, Similarity: 0.25, Template Neff: 5.9

```
Q ss_pred              HHHHHHHHHHHhhchHHHHHHHHHHHHHHHHHHHHHHHHHHHHHHHHHHHHHHHHHhHHHHHHHHHHHHHHHHHHHh--
Q Q_1957385    250    TQLMATFIQLVGKNNEESLKNDLALFQSLQESRKTEMERKSDEYAAEVRKAEELNRVMGCVGKILGALLTIVSVVAAA--   327 (580)
Q Consensus    250    ~~Lma~l~~l~~~~~~~~~~l~~~~e~~~~~~~~a~~~~~~kk~eE~~e~~~KAe~~~K~~GcigKIlG~i~~avsvvaav--   327 (580)
                       ..+...|..+.++..+.+.+...+.++..++.++++++++.++++|..+|+++++|. |||+||||||..++++|+++
T Consensus     58    ~~l~~~L~~L~~kl~~~q~~~~~~~I~~~~~~~~~~~~~~e~~~Ki~Ea~ek~~eAkK~-G~~~KIfgwI~a~~svV~gav~   136 (219)
T 3WXX_B        58    EEFEIELSKMTSELEQTQKKLKLADLERIRAENLKKIDENQTKMKEASEAADKAKKS-GLASKIFGWISAIASMVIGAIL   136 (219)
T ss_dssp              CCCCC----------------CHHHHHHHHHHTHHHHHHHHHHHHHHHHHHHHHHHS-SCHHHHSTTSCHHHHHHHHHHH
T ss_pred              HHHHHHHHHHHHHHHHHHHHHHHHHHHHHHHHHHHHHHHHHHHHHHHHHHHHHHHHc-cHHHHHHHHHHHHHHHHHHHHH

Q ss_pred              -ccHH--HHHHHHHHHHHHHHHHHHHHHhCCCHHHHHHHHHHHHHHHHHHHHHHHHHHHHHHHCCCCHHHHHHHHHHHHH
Q Q_1957385    328    -FSGG--ASLALAAVGLALMVTDAIVQAATGNSFMEQALNPIMKAVIEPLIKLLSDAFTKMLEGLGVDSKKAKMIGSILG   404 (580)
Q Consensus    328    -fTGG--aslalAavglal~vaD~I~~a~TG~s~~~~~m~PlM~~v~~pl~~~i~~~i~~~L~~~Gvd~~~A~~ig~I~~   404 (580)
                        +|||  ++-++...+.++++++.++++++|.++                                ++++..+.++.+..
T Consensus    137    vatG~ga~Aga~liA~gvi~vv~~iv~~a~~~g~--------------------------------i~~~~~k~~G~v~~   184 (219)
T 3WXX_B       137    IATGVGAAVGAMMIVGGAVGVANMAIQQAAADGR--------------------------------ISPETMKVLGPIMI   184 (219)
T ss_dssp              HHHHHHHHHHHHHHHCTTTHHHHHHCCC-----------------------------------------CCCCSHHHHHH
T ss_pred              HHccchHHHHHHHHHHHHHHHHHHHHHHHhcccc--------------------------------CCHHHHHHhhHHHH

Q ss_pred              HHHHHHHHHHHHHHHHHHHHHHHHHHHHHHHH
Q Q_1957385    405    AIAGALVLVAAVVLVATVGKQAAAKLAENIGK   436 (580)
Q Consensus    405    av~~ava~va~~~~~~~~~~~aa~~~~~~~a~   436 (580)
                       ++-.+++++.+++..+....+.+++..+..++
T Consensus    185    ~~~I~~avv~~~~s~g~~aa~~~~~~~~k~a~~   216 (219)
T 3WXX_B       185    AAEILVAIVSIAVTFGASAASTAMKAVKFATQ   216 (219)
T ss_dssp              HHHHHHHHHHHHHHSCHHHHHHHHHHHHHHHH
T ss_pred              HHHHHHHHHHHHHHHhHHHHHHHHHHHHHHHh
```

Supplementary Figure 2. Remote protein homology between AopB and the IpaB domain following the long Coiled Coil domain of IpaB is detected with various fold recognition servers. Here, *Aeromonas hydrophila* AopB sequence (PDB id 3WXX, chain B) (Nguyen et al., 2015) is shown in pair-wise alignment with the domain of IpaB (residues 250 to 435) as an output of the HHpred prediction server (Zimmermann et al., 2018). The IpaB sequence can be modeled after the AopB structural template with a 99.51% confidence despite the low sequence identity (19%).

2. Supplementary Tables

Calc. Mass	Obsrv. Mass	± da	± ppm	Start Seq.	End Seq.	Sequence	Ion Score	C. I. %
872,4948	872,5002	0,0054	6	158	164	INQIQTR		
1167,5641	1167,569	0,0045	4	290	299	SDEYAAEVRK		
1196,6633	1196,604	-0,0591	-49	425	436	QAAAKLAENIGK		
1329,7737	1329,772	-0,0018	-1	178	188	LSREEIQLTIK	51	99,765
1329,7737	1329,772	-0,0018	-1	178	188	LSREEIQLTIK		
1446,7799	1446,779	-0,0012	-1	123	135	INTLLSETEGLTR	109	100
1446,7799	1446,779	-0,0012	-1	123	135	INTLLSETEGLTR		
1520,7704	1520,768	-0,0022	-1	270	282	NDLALFQSLQESR	134	100
1520,7704	1520,768	-0,0022	-1	270	282	NDLALFQSLQESR		
1604,8101	1604,799	-0,0113	-7	1	15	MHNVSTTTTGFPLAK		
2160,0779	2160,068	-0,0098	-5	16	36	ILTSTELGDNTIQAANDAANK	135	100
2160,0779	2160,068	-0,0098	-5	16	36	ILTSTELGDNTIQAANDAANK		
2280,1719	2280,182	0,0102	4	116	135	NLEFSDKINTLLSETEGLTR	149	100
2280,1719	2280,182	0,0102	4	116	135	NLEFSDKINTLLSETEGLTR		
2335,1523	2335,164	0,0118	5	263	282	NNEESLKNDLALFQSLQESR	205	100
2335,1523	2335,164	0,0118	5	263	282	NNEESLKNDLALFQSLQESR		
2371,1047	2371,132	0,0269	11	220	241	EIDSFSAFSNTASAEQLSTQQK		
2389,1155	2389,108	-0,0071	-3	220	241	EIDSFSAFSNTASAEQLSTQQK		
2392,1926	2392,162	-0,031	-13	546	567	FGQLQEVIADLLASMSNSQANR	11	0
2392,1926	2392,162	-0,031	-13	546	567	FGQLQEVIADLLASMSNSQANR		

Supplementary Table 1. Mass spectrometry analysis performed on the IpaB-N band isolated from the SDS-PAGE gel of Supplementary Figure 1, lane 3. Information of the peptides detected is presented. The analysis was performed by the Mass Spec facility (ProteoPole) of Institut Pasteur.

Model	Molecule	PDB template	PDB template residue numbers	Model residue numbers*
Asymmetric IpgC	IpgC	3GZ1	8-151	8-151
Symmetric IpgC	IpgC	3KS2	18-154	18-154
IpgC-IpaB dimer	IpgC (AcrH)	3KS2 (3WXX)	30-154 (10-159)	30-154 (13-154)
	IpaB	3GZ1	60-72	60-72
	IpaB	5WKQ	74-239	74-239
	IpaB (AopB)	3WXX	71-263	246-438

Supplementary Table 2. PDB models used for the modeling

*The numbering of the wild type protein is employed in the table but the IpgC construct we used contains the N-terminal tag "MGSSHHHHHHSSGLVPRG" (+17 residues).

3. Supplementary Video

Supplementary Video 1. Three-dimensional view of the IpgC/IpaB complex SAXS model.

4. References

Nguyen, V. S., Jobichen, C., Tan, K. W., Tan, Y. W., Chan, S. L., Ramesh, K., et al. (2015). Structure of AcrH-AopB Chaperone-Translocator Complex Reveals a Role for Membrane Hairpins in Type III Secretion System Translocon Assembly. *Structure* 23, 2022–2031. doi:10.1016/j.str.2015.08.014.

Zimmermann, L., Stephens, A., Nam, S. Z., Rau, D., Kübler, J., Lozajic, M., et al. (2018). A Completely Reimplemented MPI Bioinformatics Toolkit with a New HHpred Server at its Core. *J. Mol. Biol.* 430, 2237–2243. doi:10.1016/j.jmb.2017.12.007.
